# Survival analysis of patients with extrahepatic cholangiocarcinoma: a nomogram for clinical and MRI features

**DOI:** 10.1186/s12880-023-01188-y

**Published:** 2024-01-02

**Authors:** Yanyan Zeng, Xiaoyong Wang, Jiaojiao Wu, Limin Wang, Feng Shi, Jian Shu

**Affiliations:** 1 Department of Radiology, The Affiliated Hospital of Southwest Medical University, 25 Taiping Street, 646000 Luzhou, China; 2 Department of Research and Development, Shanghai United Imaging Intelligence Co., Ltd, 200030 Shanghai, China

**Keywords:** Survival analysis, Nomogram, Magnetic resonance imaging, Extrahepatic cholangiocarcinoma

## Abstract

**Background:**

This study aimed to establish a predictive model to estimate the postoperative prognosis of patients with extrahepatic cholangiocarcinoma (ECC) based on preoperative clinical and MRI features.

**Methods:**

A total of 104 patients with ECC confirmed by surgery and pathology were enrolled from January 2013 to July 2021, whose preoperative clinical, laboratory, and MRI data were retrospectively collected and examined, and the effects of clinical and imaging characteristics on overall survival (OS) were analyzed by constructing Cox proportional hazard regression models. A nomogram was constructed to predict OS, and calibration curves and time-dependent receiver operating characteristic (ROC) curves were employed to assess OS accuracy.

**Results:**

Multivariate regression analyses revealed that gender, DBIL, ALT, GGT, tumor size, lesion’s position, the signal intensity ratio of liver to paraspinal muscle (SIR_Liver/Muscle_), and the signal intensity ratio of spleen to paraspinal muscle (SIR_Spleen/Muscle_) on T2WI sequences were significantly associated with OS, and these variables were included in a nomogram. The concordance index of nomogram for predicting OS was 0.766, and the AUC values of the nomogram predicting 1-year and 2-year OS rates were 0.838 and 0.863, respectively. The calibration curve demonstrated good agreement between predicted and observed OS. 5-fold and 10-fold cross-validation show good stability of nomogram predictions.

**Conclusions:**

Our nomogram based on clinical, laboratory, and MRI features well predicted OS of ECC patients, and could be considered as a convenient and personalized prediction tool for clinicians to make decisions.

**Supplementary Information:**

The online version contains supplementary material available at 10.1186/s12880-023-01188-y.

## Background

Cholangiocarcinoma (CCA) is a fatal hepatobiliary adenocarcinoma with a high mortality rate and poor prognosis, accounts for almost 3% of all gastrointestinal malignancies [1]. Extrahepatic cholangiocarcinoma (ECC) is highly invasive, difficult to diagnose early, and hard to surgically remove due to its complex anatomy. As a result, patients has 5-year relative survival rates ranging from 2 to 30% [2]. While surgical resection is the primary treatment option for ECC patients, the recurrence rate remains disappointingly high [3, 4]. In this context, vigilant postoperative monitoring is crucial for assessing treatment efficacy and the potential for recurrence [5]. However, patients who have not received appropriate treatment may be subjected to excessive or inadequate treatment, causing unnecessary side effects [6]. Given these challenges, he preoperative identification of prognostic biomarkers is imperative to assist patients in selecting the most appropriate treatment strategy prior to the commencement of therapy, thereby enhancing the likelihood of a favorable prognosis. Based on this, constructing a predictive model using preoperative biomarkers can provide a basis for clinicians to develop personalized treatment strategies.

Tumor-node-metastasis (TNM) staging serves as a valuable reference for clinicians in prognostic assessments and treatment planning [7]. However, the TNM staging system solely considers tumor size, depth, and lymph node involvement, disregarding the overall patient condition and other prognostic factors. Consequently, it fails to comprehensively depict the biological behavior and prognosis of tumors, resulting in disparate prognoses for certain tumors within the same stage while others may exhibit similar prognoses across different stages [8]. Therefore, relying solely on three criteria—tumor size, lymph node metastasis, and distant metastasis—to judge prognosis is imprecise and inaccurate. As a non-invasive diagnostic tool, MRI has been widely used in the diagnosis and treatment of ECC [9]. MRI can provide high-resolution images that can display the location, shape, size, and infiltration range of ECC, which plays an important role in preoperative evaluation, selection of surgical plans, and evaluation of treatment effects and recurrence [10]. Additionally, MRI can also be utilized for assessing the correlation between lesions of bile duct cancer and adjacent tissues or vital structures. By comparing the discrepancies in MRI signals between normal surrounding tissues of the bile duct and those surrounding the lesion of cholangiocarcinoma, it becomes feasible to determine whether there is infiltration into the neighboring tissue by the lesion, thereby aiding in evaluating the extent of tumor invasion and metastasis [10, 11]. In recent years, the utility of MRI as a prognostic tool for postoperative survival in ECC patients has increasingly come into focus [12, 13].

MRI is a crucial modality for assessing signal alterations in the liver and spleen. Research has demonstrated a close association between abnormal liver MRI signals and liver injury, iron overload, and anemia [14]. Specifically, the intensity of splenic signals on MRI has been shown to have a substantial association with serum ferritin levels, reflecting the severity of anemia. Iron overload can result in impaired organ function, potentially leading to organ failure [15]. Moreover, certain biomarkers indicative of liver injury and cholestasis, such as gamma-glutamyl transferase (GGT), alanine aminotransferase (ALT), aspartate aminotransferase (AST), and total bilirubin (TBIL) have been utilized to reflect the status of hepatic damage and evaluate prognostic outcomes in patients with cholangiocarcinoma.

In general, this study endeavors to formulate a preoperative prognostic model, integrating preoperative laboratory analyses and MRI characteristics, to precisely forecast the prognosis for ECC patients undergoing curative resection.

## Materials and methods

### Patients

This study was conducted in strict adherence to the principles set forth in the “Declaration of Helsinki.” The Institutional Review Board in The Affiliated Hospital of Southwest Medical University granted approval for this retrospective study, and the requirement for written informed consent from the patients was waived in accordance with the regulations. To ensure the confidentiality of patient information, all identifying details were meticulously removed from the records. Patients with ECC diagnosed by postoperative pathology at our hospital from January 2013 to July 2021 were included. The inclusion criteria mandated that patients (1) had undergone radical resection or pancreaticoduodenectomy, (2) were free of other malignant neoplasms or intrahepatic metastases, (3) had no prior exposure to anticancer treatments like radiotherapy or chemotherapy before surgery, and (4) had undergone an MRI scan within one month prior to surgery. Conversely, exclusion criteria included (1) death during the perioperative period; (2) incomplete clinical and imaging data; and (3) lesions smaller than 5 mm and undetectable in MRI. Ultimately, a total of 104 patients satisfying the stringent criteria were enrolled in this study (Supplementary Fig. 1). To ensure accurate and standardized pathological determinations, an experienced abdominal pathologist with a decade of diagnostic expertise meticulously reviewed the pathological assessments of ECC. In addition to the pathological evaluation, various clinical parameters were taken into consideration, encompassing gender, age, ALT, AST, TBIL, direct bilirubin (DBIL), GGT and Carbohydrate antigen199 (CA199).

### MRI scan

The MRI images of all patients included in this study were obtained using a 3.0T MRI scanner (Acthia3.0T, Philips) and a 16-channel abdominal coil. All patients were instructed to fasting for 6-8 h before examination, and were requested to practice breathing and breath holding. The scan range was from the upper edge of the liver to the descending segment of the duodenum. The following MRI sequences were collected: axial fat-suppression turbo spin echo (TSE) T2-weighted imaging (T2WI) sequence and axial diffusion-weighted imaging (DWI). This study mainly aimed at analyzing the imaging features of T1WI, T2WI, DWI sequences, and the apparent diffusion coefficient (ADC) maps. The parameters of some MRI sequences are presented in Supplementary Table 1.

The ADC maps were automatically derived from DWI (b = 0 and 800 s/mm^2^) images in Extended MR Workspace R2.6.3.1 (Philip Healthcare). The signal intensity of the lesion on the DWI (b = 800 s/mm^2^) sequence was measured, and the ADC values for the lesion (SI_lesion_) on the ADC map were similarly determined. The ADC values were measured by copying the same regions of interest (ROI) from the DWI sequence to the ADC map. The ROI of the lesion should be near the center of the lesion and measured thrice to obtain the average. During measurement, care should be taken to avoid artifacts, blood vessels, bile ducts, and necrotic areas. The ADC values for the spleen (SI_Spleen_) and liver (SI_Liver_) were measured in the same way as those for the lesion. In addition, the signal intensity ratio of the liver-to-muscle (SIR_Liver/Muscle_) was measured using images obtained with each T2-weighted image by means of the ROI positioned in the liver and paraspinal muscle. In the same way, SIR_Spleen/Muscle_ was also determined on the T2WI sequence.

### MRI features

All the scan sequences of the patients were comprehensively observed, and the following imaging traits were collected: (1) lesion’s location (Perihilar cholangiocarcinoma (pCCA) is localized between the left/right hepatic duct and insertion of the cystic duct into the common bile duct and distal (dCCA) is confined to the common bile duct); (2) morphology (mass-forming type, periductal infiltrating type, and intraductal growth type); (3) tumor size (maximum diameter of the tumor measured on the axial T2WI images); (4) lesion’s signal (homogeneous or heterogeneous); (5) intrahepatic bile duct dilatation (diameter of the widest intrahepatic bile duct at 1 cm from the confluence of the left and right hepatic ducts was measured); (6) DWI signal (when b = 800 s/mm^2^, the lesion showed high signal or low signal); (7) SI_Lesion_; (8) SI_Spleen_; (9) SI_Liver_; (10) SIR_Liver/Muscle_; 11) SIR_Spleen/Muscle_.

### Follow-up

Patient outcomes, including survival time and cause of death, were obtained through medical records, telephone follow-up, or local population database. Overall survival (OS) was defined as the duration between the date of operation and the date of death or the last follow-up. It is noteworthy that the last follow-up for this study occurred on July 31, 2021. Within the cohort, patients were categorized into two distinct groups: the death group, comprising individuals who passed away due to ECC during the follow-up, with survival duration measured from surgery onset to death date; and the survival group, consisting of those alive at follow-up’s end, with survival time calculated from surgery commencement to the last outpatient visit. It is important to note that the survival group data was considered as censored data, allowing for a comprehensive analysis of patient outcomes, even beyond the study’s timeframe.

### Statistical analysis

Continuous variables adhering to a normal distribution were articulated as mean ± standard deviation and compared using the t-test. Conversely, variables deviating from normal distribution were represented as median and interquartile range (25th and 75th percentiles, Q1, Q3) and analyzed via the Mann-Whitney U test. Categorical variables were expressed as counts and percentages, with the chi-square test employed for comparative analysis. Statistical significance was established at a two-tailed p-value of < 0.05.

Univariate COX regression analysis was performed to identify potential risk factors for OS. Subsequent to univariate analysis, variables with *p* < 0.10 were entered into multivariate analysis. The independent variables were tested for proportional hazards assumptions and applied to model development. The Akaike information criterion was employed to choose the final predictive model. COX regression analyses were used to detect the independent prognostic factors for OS. A two-tailed *p*-value < 0.05 were considered as statistically significant in univariate or multivariate analysis. Additionally,, the hazard ratio (HR) and the associated 95% confidence interval (CI) were also calculated. In R Studio, independent risk factors were used to establish a nomogram for predicting 1- and 2-year OS. Then, the internal verification of the nomogram was performed using 2000 bootstrap iterations. Calibration curves and C-index were used to evaluate the precision of the nomogram, and ROC curves were used to evaluate the accuracy of the nomogram’s 1- and 2-year forecasts. Besides, k-fold cross-validation was used to verify the stability of the nomogram in predicting OS. All statistical analyses were implemented using SPSS (version 26.0) and R software (version 4.1.2, https://www.rproject.org). To evaluate and validate the reliability of the measurement data, a subset of 20 patients was selected from a total cohort of 104 patients. Two experienced radiologists were invited to assess the MRI features across T1WI, T2WI, and DWI in order to compare inter-observer consistency. By calculating the intraclass correlation coefficient (ICC) or Kappa values for each feature among observers, we confirmed the reliability of acquiring MRI feature data. Notably, an ICC or Kappa values ≥ 0.7 indicated excellent agreement among observers.

## Results

### Intra-observer agreement

Based on the intra-observer reliability assessment performed by two radiologists, all MRI features exhibited ICC or Kappa values exceeding 0.7 (*P <* 0.05).

### Patients characteristics

Among 104 patients who met the inclusion criteria for ECC, 64 and 40 patients died and survived at the last follow-up, respectively. The estimated median survival time was 18 months (95% CI: 14.9–21.1). The postoperative 1-year and 2-year estimated OS rates were 62.2% and 39.8%, respectively. Detailed clinicopathological characteristics of ECC patients were summarized in Supplementary Table 2. When comparing the distribution of nine characteristics between the death and survival groups, there were significant differences in follow-up time, gender, TBIL, and DBIL values(*p* < 0.05). Median follow-up times were 8.5 and 34 months, respectively. Female representation was 34 (53.1%) and 12 (30%) in each cohort. Median TBIL was noted as 175.9 U/L and 85.8 U/L, while DBIL was 139.9 U/L and 66.9 U/L, respectively. There were no significant differences between these two groups in terms of age (*p* = 0.973), ALT (*p* = 0.498), AST (*p* = 0.44), GGT (*p* = 0.506), and CA199 (*p* = 0.123). Utilizing X-tile 3.6.1 (https://x-tile.software.informer.com/), continuous variables were dichotomized for enhanced clinical applicability. Optimal thresholds for each variable were established, updating corresponding statistics in Table 1. As observed, there were statistically significant differences in ALT (> 68.4 U/L), TBIL (> 122.5 µmol/L), DBIL (> 93.9 µmol/L), and CA199 (> 197.0 U/mL) between death and survival groups.

**Table 1:** Binarized clinicopathological characteristics of ECC patients in death and survival groups

Binarized characteristics	All samples (n = 104)	Death (n = 64)	Survival (n = 40)	*p* value
Time (month)	13.5 (6.0, 31.8)	8.5 (4.0, 17.8)	34.0 (11.5, 60.3)	< 0.001
Age (≥ 62 years old)	53 (51.0%)	34 (53.1%)	19 (47.5%)	0.577
Gender (female)	46 (44.2%)	34 (53.1%)	12 (30.0%)	0.021
ALT (> 68.4 U/L)	65 (62.5%)	45 (70.3%)	20 (50.0%)	0.037
AST (> 51.4 U/L)	77 (74.0%)	49 (76.6%)	28 (70.0%)	0.458
TBIL (> 122.5 µmol/L)	63 (60.6%)	49 (76.6%)	14 (35.0%)	< 0.001
DBIL (> 93.9 µmol/L)	67 (64.4%)	52 (81.3%)	15 (37.5%)	< 0.001
GGT (> 600.2 U/L)	39 (37.5%)	20 (31.3%)	19 (47.5%)	0.096
CA199 (> 197.0 U/mL)	33 (31.7%)	25 (39.1%)	8 (20.0%)	0.042

**Fig. 1:**
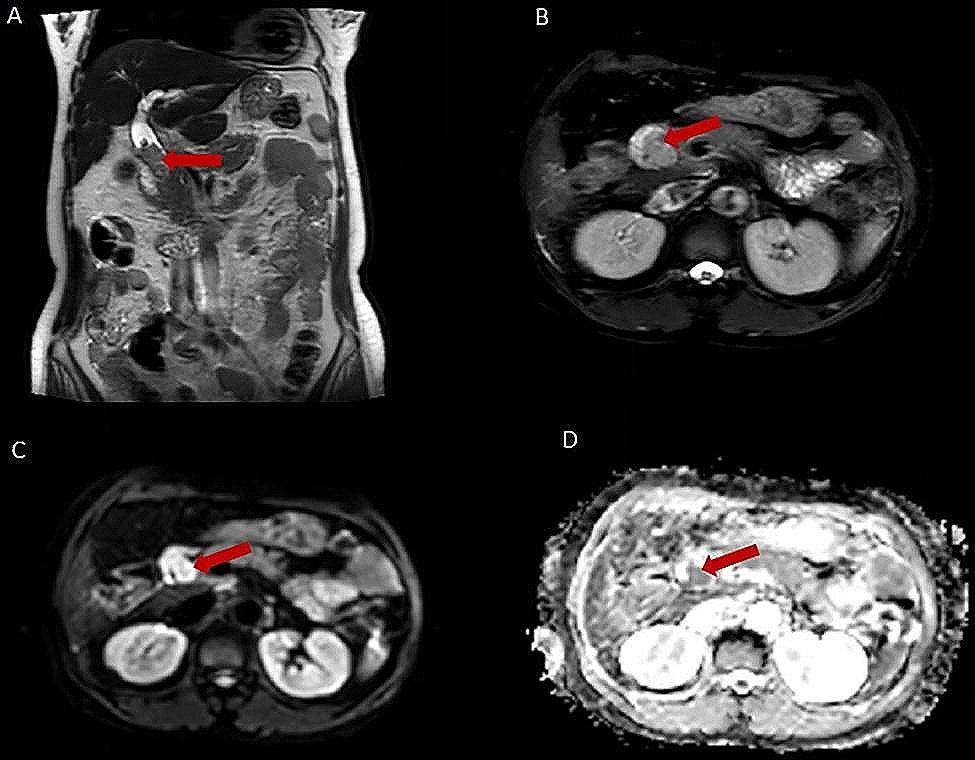
MRI image of a patient with distal cholangiocarcinoma. (**A**) and (**B**) T2-weighted images showing the lesion’s location, size, morphology, and MRI signal characteristics. (**C**) Diffusion-weighted images showing a intermediate/high signal for the tumor. (**D**) Apparent diffusion coefficient (ADC) map showing a low signal for the tumor. The red arrows pointed to the tumor

Additionally, all MRI features are extracted in the T2WI and DWI images. Representative images were displayed in Fig. 1, in which the lesion’s location, size, morphology, and MRI signal characteristics could be observed by T2WI (Fig. 1a and b), and a intermediate/high signal and a low signal could be shown in the DWI (Fig. 1c) and apparent diffusion coefficient (ADC) map (Fig. 1d), respectively. We extracted and compared some features from these images that reflect the malignancy itself, as well as some indirect MRI features that may affect the patients’ prognosis. As shown in Supplementary Table 3, the morphology, especially the mass-forming type, was statistically different between the death and survival groups. Mass-forming type was detected in 16 (25%) patients in the death group and only 1 (2.5%) patient in the survival group (*p* = 0.010). Furthermore, Tumor size (*p* = 0.094), lesion’s location (*p* = 0.253), lesion’s signal (*p* = 0.850), intrahepatic bile duct dilatation (*p* = 0.981), DWI signal (*p* = 0.397), ADC_Lesion_ (*p* = 0.411), SIR_Liver/Muscle_ (*p* = 0.434), SIR_Spleen/Muscle_ (*p* = 0.326), SI_Liver_ (*p* = 0.963), SI_Spleen_ (*p* = 0.063) were no significant differences between these two groups. Similarly, some of the continuous variables were binarized to improve the clinical utility of MRI features in predicting OS. Table 2 summarizes the optimal cut-off values and corresponding statistics for these variables. Statistically significant differences were observed in tumor size (> 3.2 cm) and morphology between the death and survival groups.

**Table 2:** Binarized MRI features of ECC patients in death and survival groups

Imaging features	All samples (n = 104)	Death (n = 64)	Survival (n = 40)	*p* value
Tumor size (> 3.2 cm)	18 (17.3%)	16 (25.0%)	2 (5.0%)	0.009
Lesion’s location (dCCA)	63 (60.6%)	36 (56.3%)	27 (67.5%)	0.253
Morphology				0.010
- Periductal infiltrating type	44 (42.3%)	25 (39.1%)	19 (47.5%)	
- Intraductal growth type	43 (41.3%)	23 (35.9%)	20 (50.0%)	
- Mass-forming type	17 (16.3%)	16 (25.0%)	1 (2.5%)	
Lesion’s signal (homogeneous)	43 (41.3%)	26 (40.6%)	17 (42.5%)	0.850
Intrahepatic bile duct dilatation (> 1.2 cm)	52 (50.0%)	33 (51.6%)	19 (47.5%)	0.687
DWI signal (high)	80 (76.9%)	51 (79.7%)	29 (72.5%)	0.397
ADC_Lesion_ (> 1.5 mm^2^/s)	21 (20.2%)	11 (17.2%)	10 (25.0%)	0.334
SIR_Liver/Muscle_ (> 0.7)	89 (85.6%)	53 (82.8%)	36 (90.0%)	0.310
SIR_Spleen/Muscle_ (> 1.9)	61 (58.7%)	34 (53.1%)	27 (67.5%)	0.148
SI_Liver_ (> 1.0)	57 (54.8%)	35 (54.7%)	22 (55.0%)	0.975
SI_Spleen_ (> 0.8)	69 (66.3%)	46 (71.9%)	23 (57.5%)	0.131

### Univariate and multivariate analyses of risk factors on OS

To identify the most important factors on OS, a COX proportional hazards regression model was developed through sequential univariate and stepwise multivariate COX regression analyses (Table 3**)**. As shown in Table 3 and Supplementary Fig. 2, female (adjusted HR = 2.27 [1.30–3.94], *p* = 0.004), ALT > 68.4 U/L (adjusted HR = 1.88 [1.02–3.45], *p* = 0.043), DBIL > 93.9 µmol/L (adjusted HR = 4.23 [2.20–8.13], *p* < 0.001), tumor size > 3.2 cm (adjusted HR = 2.53 [1.40–4.60], *p* = 0.002), dCCA (adjusted HR = 0.49 [0.28–0.85], *p* = 0.011), GGT > 600.2 U/L (adjusted HR = 0.53 [0.29–0.97], *p* = 0.039), SIR_Liver/Muscle_ > 0.7 (adjusted HR = 0.41 [0.20–0.83], *p* = 0.014), and SIR_Spleen/Muscle_ > 1.9 (adjusted HR = 0.51 [0.29–0.88], *p* = 0.016) were strongly associated with OS. Kaplan-Meier survival curves and corresponding Log Rank tests (Supplementary Fig.3)corroborated the significance of these eight factors in OS, aligning with Cox regression outcomes. Patients with female, DBIL > 93.9 µmol/L, ALT > 68.4 U/L, and GGT ≤ 600.2U/L had a worse prognosis among clinical features, while patients with pCCA, tumor size > 3.2 cm, SIR_Liver/Muscle_ ≤ 0.7, and SIR_Spleen/Muscle_ ≤ 1.9 had a lower overall postoperative survival among MRI features. Notably, other clinical indicators and imaging features were not statistically significant (*p* > 0.05).

**Table 3:** Univariate and multivariate analyses of OS for patients with ECC. *p* < 0.05 were considered as statistically significant in univariate or multivariate analysis

Characteristics		Univariable analysis		Multivariable analysis	
		HR (95% CI)	*p* value	Adjusted HR (95% CI)	*p* value
Clinicopathological	Gender (female)	1.83 (1.11–3.00)	0.017	2.27 (1.30–3.94)	0.004
	ALT (> 68.4 U/L)	1.90 (1.11–3.25)	0.020	1.88 (1.02–3.45)	0.043
	TBIL (> 122.5 µmol/L)	3.01 (1.68–5.37)	< 0.001		
	DBIL (> 93.9 µmol/L)	3.51 (1.87–6.59)	< 0.001	4.23 (2.20–8.13)	< 0.001
	GGT (> 600.2 U/L)	0.58 (0.34–0.98)	0.044	0.53 (0.29–0.97)	0.039
	CA199 (> 197.0 U/mL)	1.88 (1.13–3.13)	0.015		
Imaging	Tumor size (> 3.2 cm)	2.19 (1.23–3.88)	0.008	2.53 (1.40–4.60)	0.002
	Lesion’s location (dCCA)	0.64 (0.39–1.05)	0.077	0.49 (0.28–0.85)	0.011
	Morphology (intraductal growth type)	0.75 (0.43–1.03)	0.330		
	Morphology (mass-forming type)	1.95 (1.03–3.68)	0.039		
	SIR_Liver/Muscle_ (> 0.7)	0.45 (0.23–0.87)	0.018	0.41 (0.20–0.83)	0.014
	SIR_Spleen/Muscle_ (> 1.9)	0.49 (0.30–0.81)	0.005	0.51 (0.29–0.88)	0.016

### Nomograms construction for predicting OS

**Fig. 2:**
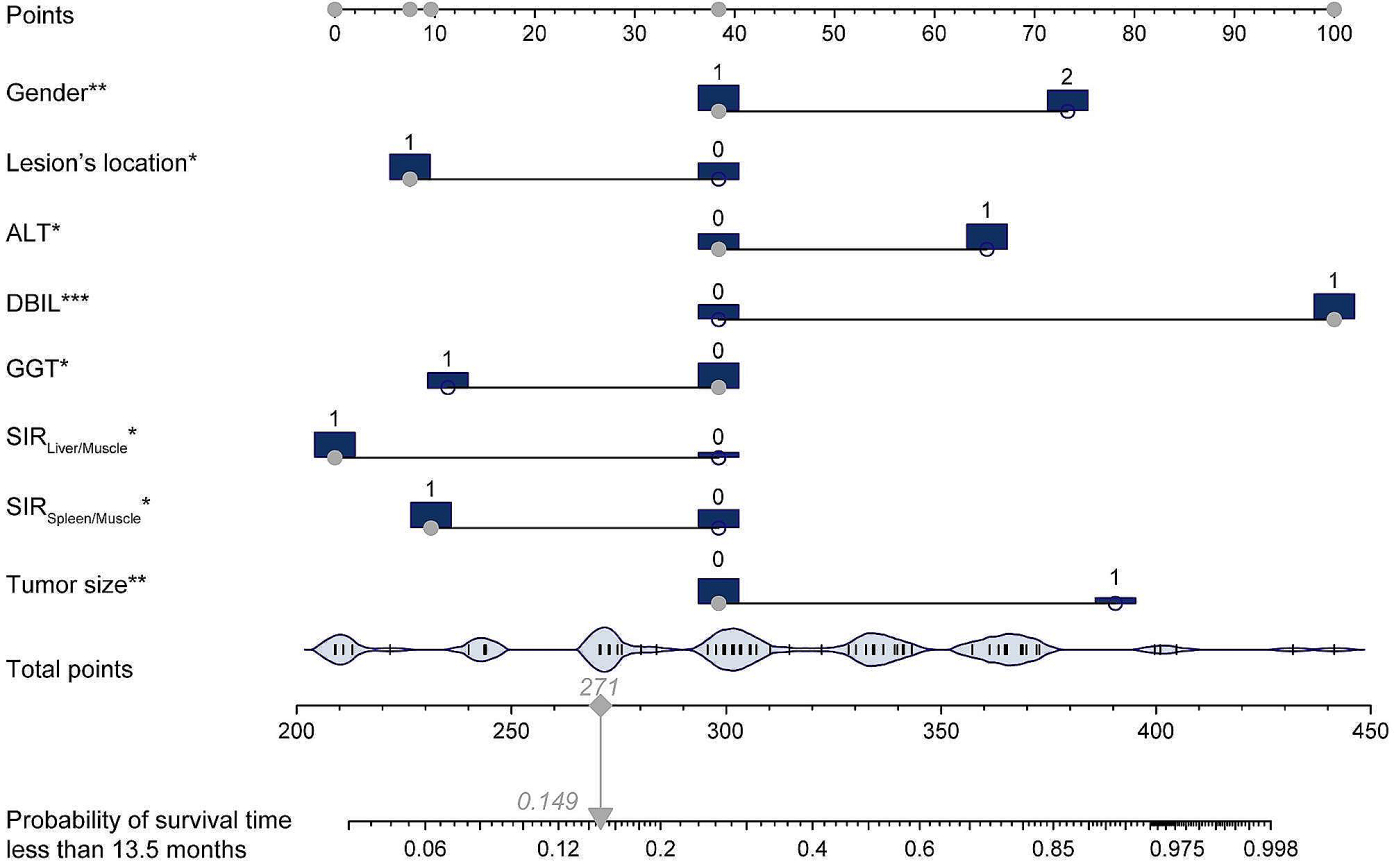
The nomogram composed of eight variables predicting the probability of survival time less than the median follow-up time (13.5 months). For a given sample, each variable had a point, and the total point reflected the final probability. The blue bars and bean charts represented the distribution of patients corresponding to the different characteristics. Asterisks represented the statistical results of multivariate COX regression, with * indicating *p* < 0.05, ** indicating *p* < 0.01, and *** indicating *p* < 0.001

**Fig. 3:**
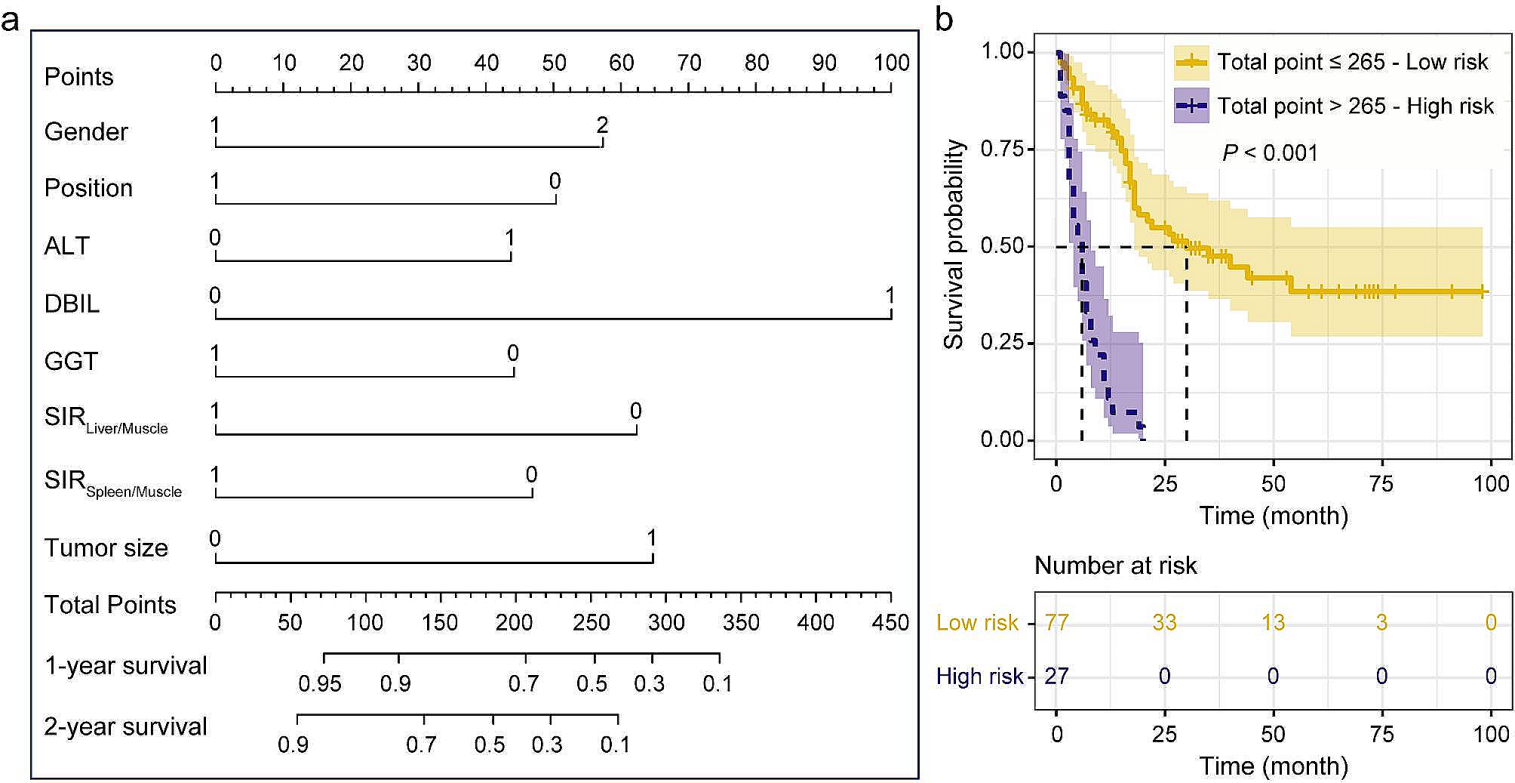
The nomogram assessing the risk probability of the ECC patient after treatment. (**a**) Nomogram composed of eight variables predicting 1-year and 2-year survival probability. For a given sample, each variable had a point, and the total point corresponded to the survival probability at a specific time. (**b**) Kaplan-Meier survival curve showing that the total point calculated from the nomogram well defined the risk levels with significantly different survival times

Utilizing the chosen eight variables, the optimal Cox proportional hazard model was established (*χ*^2^ = 61.3, *p* < 0.001). Within this framework, the risk score was conceptualized as the natural logarithm of the ratio of the hazard function relative to the baseline hazard function. The corresponding formula is delineated as follows:

$$ \begin{aligned}\\&\text{r}\text{i}\text{s}\text{k} \text{s}\text{c}\text{o}\text{r}\text{e}=0.818{x}_{1}+0.629{x}_{2}+1.443{x}_{3}-0.635{x}_{4}\\&\quad\quad\quad\quad+0.930{x}_{5}-0.723{x}_{6}-0.899{x}_{7}-0.674{x}_{8}\end{aligned}$$

where $$ {x}_{1}$$ denoted the gender (female), $$ {x}_{2}$$ denoted the ALT (> 68.4 U/L), $$ {x}_{3}$$ denoted the DBIL (> 93.9 µmol/L), $$ {x}_{4}$$ denoted the GGT (> 600.2 U/L), $$ {x}_{5}$$ denoted the tumor size (> 3.2 cm), $$ {x}_{6}$$ denoted the lesion’s location (dCCA), $$ {x}_{7}$$ denoted the SIR_Liver/Muscle_ (> 0.7), and $$ {x}_{8}$$ denoted the SIR_Spleen/Muscle_ (> 1.9). The concordance index (C-index) of this model was 0.766 with a standard error of 0.032, demonstrating good model fitness (Supplementary Fig.4). To specifically predict the prognosis of a patient diagnosed with ECC, two nomograms were constructed (Figs. 2 and 3), in which one was to predict the probability of survival time less than the median follow-up time (13.5 months). In this nomogram, physicians can determine the corresponding point for each variable by inputting them into the nomogram, enabling them to calculate a total point that corresponds to the respective predictive probability. Hence, physicians can promptly evaluate patients’ prognostic outcomes through the substitution of variables, facilitating the formulation of personalized treatment strategies.

To further explore the risk probability of the patient after treatment, the second nomogram was established to visualize the survival probability at a specific time, including 1 year and 2 years (Fig. 3a). For each patient, points are assigned to every variable, with the aggregate of these points across eight variables indicative of 1-year and 2-year survival probabilities. Additionally, the total point for each individual can be computed by inputting the values of predictive variables, followed by employing X-tile software to categorize patients’ total point into two distinct groups. The results show that the optimal cutoff value is 265, segregating patients into high-risk and low-risk groups. Patients in the high-risk group, characterized by an elevated prognostic risk, require more intensive monitoring and treatment strategies. Conversely, the low-risk group, associated with a reduced prognostic risk, may necessitate less rigorous monitoring and treatment approaches. From Fig. 3b, Based on the Kaplan-Meier survival analysis curve, patients classified in the high-risk group exhibited a survival duration of less than 25 months, whereas those categorized in the low-risk group demonstrated prolonged survival periods.

### Performance evaluation of the nomogram

**Fig. 4:**
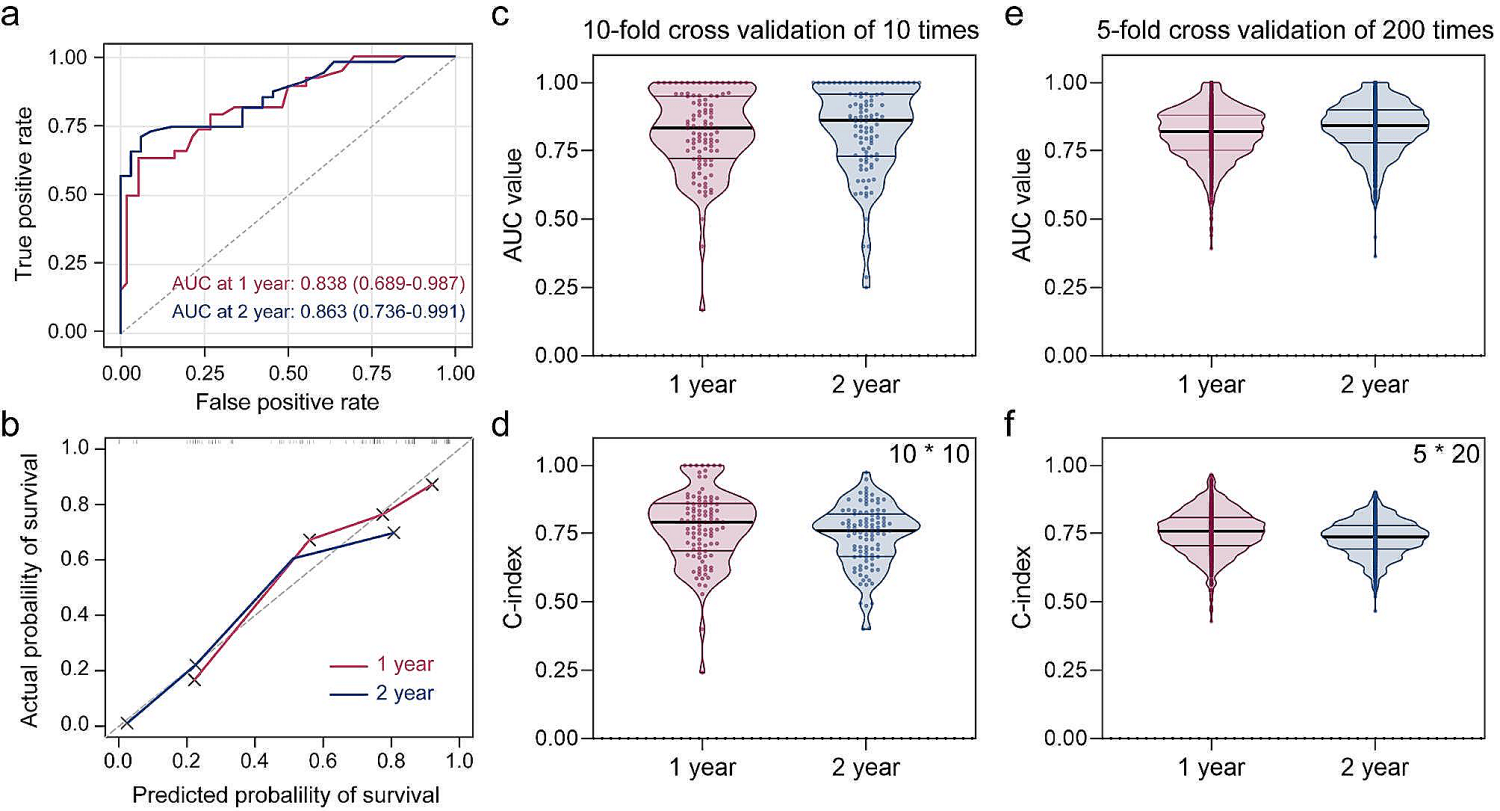
Performance evaluation of the nomogram. (**a**) Time-dependent ROC curves for 1-year and 2-year overall survival (OS) predictions. (**b**) Calibration curves for 1-year and 2-year OS predictions. (**c**) Area under the curve (AUC) value and (**d**)concordance index (C-index) of the nomogram in predicting 1-year and 2-year OS in ten-fold cross-validation experiments. (**e**) Area under the curve (AUC) value and (**f**)concordance index (C-index) of the nomogram in predicting 1-year and 2-year OS in five-fold cross-validation experiments

The model’s performance was evaluated using the concordance index (C-index), which assesses discrimination capability, ranging from 0.5 (no discrimination) to 1.0 (optimal performance). Additionally, the accuracy of model predictions was appraised using the area under the time-dependent receiver operating characteristic (ROC) curves (AUC), as depicted in Fig. 4. As shown in Fig. 4a, time-dependent ROC curves demonstrated that nomogram owned good survival predictions at 1 year and 2 years post-intervention, in which the area under the curve (AUC) values were 0.838 (0.689–0.987) and 0.863 (0.736–0.991), respectively. Also, the C-index of the nomogram for these two time points was 0.772 and 0.757 referred to Supplementary Fig. 4. In addition, 1-year and 2-year calibration curves confirmed the good agreement of the nomogram between predicted and observed outcomes (Fig. 4b**)**. A ten-fold cross-validation of 10 times and a five-fold cross-validation of 200 times were also used to verify the stable performance of the nomogram in predicting status at a given time. Specifically, the results of 10-fold cross-validation were shown in Fig. 4c and d and Supplementary Table 4, where the AUC values for 1-year and 2-year survival predictions were 0.833 (0.719, 0.951) and 0.863 (0.731, 0.952), respectively, with corresponding C-indexes of 0.786 (0.683, 0.861) and 0.755 (0.666, 0.818). Similar results were also found in the 5-fold cross-validation (Fig. 4e and f). It should be noted that the performance of the latter was slightly weaker than the former, probably due to more repetitions and therefore more consistent results. Overall, the nomogram, consisting of eight important variables, possessed good performance in predicting the survival probability at specific times and could be considered as a personalized prediction tool for clinical decision-making.

## Discussion

ECC, a relatively uncommon biliary tract malignancy, is characterized by its insidious onset, complicating early diagnosis and often leading to advanced-stage presentations. This typically impedes surgical resection in most cases, contributing to a dismal prognosis [16], thereby highlighting the imperative for early detection. Consequently, precise prognostication of ECC patient outcomes is vital for guiding treatment strategies and enhancing quality of life. Our research introduces a novel nomogram that combines preoperative clinical variables and traditional imaging features to predict prognosis. Distinct from radiomics approaches, this nomogram allows clinicians to estimate predictive probabilities without the need for specialized software or region-of-interest (ROI) delineation, simply by evaluating traditional imaging features. This enables quick assessments based on each patient’s specific situation. Our study identified key prognostic indicators, including gender, ALT, GGT, DBIL among clinical features and tumor size, tumor location, SIR_Liver/Muscle_ ratio, SIR_Spleen/Muscle_ ratio among MRI features. Importantly, the incorporation of SIR_Liver/Muscle_ and SIR_Spleen/Muscle_ ratios as predictors represents a novel discovery not previously documented in cholangiocarcinoma prognosis studies.

Specifically, we observed a negative correlation between SIR_Liver/Muscle_ and the risk of death. When the muscle signal remains unchanged, the decrease in SIR_liver/muscle_ is caused by a decrease in liver signal. Previous studies have linked diminished T2-weighted imaging (T2WI) signal on MRI to abnormal iron deposition in the liver, a primary storage site for iron [17]. Liver cell damage leads to the leakage of stored ferritin into the bloodstream [18], markedly lowering T2WI signal intensity [19]. Additionally, malignant tumors can produce and release ferritin, leading to anemia and blood overload [20]. Research has demonstrated that tumors associated with pernicious anemia portend a poorer prognosis [21]. Thus, the decrease in SIR_liver/muscle_ indirectly reflects abnormal liver iron metabolism and pernicious anemia, implying a poor prognosis. Similarly, the spleen plays a crucial role in blood circulation, with iron transported by erythrocytes accumulating in the reticuloendothelial cells of both the liver and spleen, influencing iron metabolism [22]. In the context of anemia or blood overload due to malignancy, splenic function is also impacted. A strong correlation between ferritin levels and abnormal spleen MRI signals has been established [17]. MRI evaluation of iron overload is feasible, as it reveals a reduced spleen signal intensity relative to paraspinal muscle in T2WI sequences [23]. In our study, patients with lower SIR_Spleen/Muscle_ demonstrated a worse prognosis, possibly attributable to tumor-induced anemia. Schnitzbauer et- al discovered that preoperative anemia was an independent factor influencing CCA patients’ prognosis [21].

In alignment with extant research, tumor size and lesion location are significantly correlated with the overall survival (OS) of patients diagnosed with ECC [24]. Notably, individuals afflicted by pCCA tend to experience a more unfavorable prognosis compared to those affected by dCCA. This disparity can be attributed to the anatomical positioning of pCCA tumors around critical blood vessels and bile duct structures, rendering them more prone to infiltrating adjacent tissues and necessitating intricate hepato-biliary resection procedures [25].

In addition, this study found that indicators such as GGT, ALT, and DBIL can reflect biliary obstruction and liver damage conditions, which are closely related to the prognosis of ECC patients. According to Zhang et al., high serum bilirubin concentration affects various pathological physiological metabolic processes including decreased albumin synthesis, reduced immune function, and altered hemodynamics [26]. Complementarily, another study established an association between lower direct bilirubin levels and extended survival durations, corroborating the findings of our research [27].

In our nomogram, we have incorporated eight variables, including gender, ALT, GGT, DBIL, tumor size, tumor location, SIR_liver/muscle_ and SIR_spleen/muscle_. This convenient and practical tool enables clinical physicians to easily perform preoperative prognosis prediction through simple measurements for effective treatment decision-making. Furthermore, the accuracy and stability of the nomogram predictions were assessed through internal validation as well as 5-fold and 10-fold cross-validation in this study. The findings demonstrate that the nomogram exhibits robust stability.

This study, while insightful, is subject to certain limitations. Firstly, being a retrospective study, it is susceptible to selection bias. Secondly, the limited lesion size, MRI scan thickness, and resolution have resulted in infrequent collection of MR features of the lesions. Therefore, for future investigations, we intend to employ thin-layer MRI scans to explore the correlation between tumor characteristics and prognosis. Additionally, given the widespread integration of artificial intelligence in the medical domain, Radiomics emerges as a promising technology with immense potential for investigating the intricate relationship between radiological texture features and clinical outcomes, alongside molecular characteristics [28]. Radiomics, by extracting comprehensive quantitative data that include both visible and subvisual elements from medical images, offers a more detailed and valuable perspective than traditional visual assessments by physicians [29]. Deep learning (DL) networks, in contrast to conventional manual segmentation methods, provide accurate, objective automatic segmentation, mitigating errors and limitations inherent in manual processes [30]. This not only broadens the range of information sources relied upon by physicians for patient condition assessment beyond a singular perspective but also effectively advances the practice of precision medicine [31]. Given the aforementioned circumstances, future research will focus on expanding the sample size, conducting multicenter studies, and applying advanced deep learning techniques to improve our model’s predictive power. We also plan to integrate multidimensional data fusion methods to amalgamate information from various domains, enabling a comprehensive evaluation of patient risks. Additionally, close collaboration with expert medical teams will be sought to provide guidance for model design and optimization.

## Conclusions

In our research, we devised a nomogram integrating accessible factors to forecast the prognoses of ECC patients post-curative surgery. This nomogram exhibited commendable accuracy and clinical utility, providing essential insights for tailored clinical decision-making.

### Electronic supplementary material

Below is the link to the electronic supplementary material.

Supplementary Material 1

## Data Availability

The data that support the findings of this study are available from the corresponding author upon reasonable request.

## Abbreviations

CCA: Cholangiocarcinoma
CI: Confidence interval
dCCA: Distal cholangiocarcinoma
DBIL: Direct bilirubin
ECC: Extrahepatic cholangiocarcinoma
HR: Hazard ratio
ICC: Intrahepatic cholangiocarcinoma
NLR: The ratio of neutrophile to lymphocyte
OS: Overall survival
pCCA: Perihilar cholangiocarcinoma
ROC: Time-dependent receiver operating characteristic curves
SIR_Liver/Muscle_: The signal intensity ratio of liver to paraspinal muscle on T2WI sequence
SIR_Spleen/Muscle_: The signal intensity ratio of spleen to paraspinal muscle on T2WI sequence
SIR-LS_ADC_: The signal intensity ratio of liver-to-spleen on ADC map
SIR-LS_T2WI_: The signal intensity ratio of liver-to-spleen on T2WI sequence
SI_Lesion_: The ADC values for the lesion on the ADC map
SI_Spleen_: The ADC values for the spleen on the ADC map
SI_Liver_: The ADC values for the liver on the ADC map
TBIL: Total bilirubin
