# Supplementary material for: Survival analysis of patients with extrahepatic cholangiocarcinoma: a nomogram for clinical and MRI features
Source: BMC Med Imaging. 2024 Jan 2;24:7. doi: 10.1186/s12880-023-01188-y (PMC10763420; doi:10.1186/s12880-023-01188-y)
Supplement: Supplementary file 1 — Supplementary Material 1 [file 12880_2023_1188_MOESM1_ESM.docx]

Supplementary Material

**Supplementary Table 1 Parameters of abdominal MRI sequences.**

| Parameters | T1WI | T2WI | DWI |
| --- | --- | --- | --- |
| TR | 3.10 | 1610 | 933 |
| TE | 1.44 | 70 | 50 |
| Flip angle (degrees) | 10 | 90 | 90 |
| Matrix size (mm × mm) | 244 × 186 | 176 × 201 | 100 × 124 |
| Field of view (FOV)(mm × mm) | 280 × 305 | 280 × 305 | 280 × 305 |
| Slice thickness (mm) | 3 | 7 | 7 |
| Slice gap (mm) | -1.5 | 1 | 1 |
| Number of slices (slices) | 120 | 24 | 48 |
| Number of excitation (NEX) | 1 | 2 | 4 |
| B values (s/mm^2^) | - | - | 0, 800 |

Note: Two MRI sequences were used including the axial fat-suppressed turbo spin echo (TSE) T2-weighted imaging (T2WI) and the axial diffusion weighted imaging (DWI). Key parameters were listed below, such as repetition time (TR) and echo time (TE).

**Supplementary Table 2 Clinicopathological characteristics of ECC patients in death and survival groups.**

| Clinicopathological characteristics | All samples (n =104) | Death (n = 64) ^#^ | Survival (n = 40) ^&^ | *p* value ** |
| --- | --- | --- | --- | --- |
| Time (month) | 13.5 (6.0, 31.8) | 8.5 (4.0, 17.8) | 34.0 (11.5, 60.3) | < 0.001 |
| Age (year) | 62.0 (53.0, 66.0) | 62.0 (53.0, 65.0) | 61.0 (53.3, 66.8) | 0.973 |
| Gender (female) | 46 (44.2%) | 34 (53.1%) | 12 (30.0%) | 0.021 |
| ALT (U/L) | 86.0 (57.6, 161.6) | 91.5 (58.7, 168.2) | 72.9 (55.9, 155.5) | 0.498 |
| AST (U/L) | 77.7 (51.3, 120.3) | 84.1 (52.3, 127.4) | 65.9 (49.7, 105.4) | 0.440 |
| TBIL (μmol/L) | 154.7 (81.9, 222.6) | 175.9 (128.4, 236.2) | 85.8 (47.6, 166.4) | 0.001 |
| DBIL (μmol/L) | 123.3 (61.5, 182.3) | 139.9 (104.8, 190.1) | 66.9 (37.8, 146.4) | 0.002 |
| GGT (U/L) | 419.2 (198.0, 829.0) | 392.5 (201.9, 797.7) | 481.2 (157.4,1088.8) | 0.506 |
| CA199 (U/mL) | 123.5 (62.4, 233.6) | 142.9 (63.2, 265.2) | 107.5 (62.4, 189.6) | 0.123 |

Note: Statistical analyses were performed between the death and the survival groups. Continuous variables with abnormal distribution were represented as median (Q1, Q3) and compared with Mann-Whitney *U* tests. Categorical variables were represented as N (%) and compared with chi-square tests.

**Supplementary Table 3 Imaging features of ECC patients in death and survival groups.**

| Imaging features | All samples  (n = 104) | Death ^#^  (n = 64) | Survival ^&^  (n = 40) | *p* value |
| --- | --- | --- | --- | --- |
| Tumor size (cm) | 2.1 (1.6, 2.8) | 2.4 (1.5, 3.2) | 1.9 (1.6, 2.6) | 0.094 |
| Lesion's location (dCCA) | 63 (60.6%) | 36 (56.3%) | 27 (67.5%) | 0.253 |
| Morphology |  |  |  | 0.010 |
| - Periductal infiltrating type | 44 (42.3%) | 25 (39.1%) | 19 (47.5%) |  |
| - Intraductal growth type | 43 (41.3%) | 23 (35.9%) | 20 (50.0%) |  |
| - Mass-forming type | 17 (16.3%) | 16 (25.0%) | 1 (2.5%) |  |
| Lesion's signal (homogeneous) | 43 (41.3%) | 26 (40.6%) | 17 (42.5%) | 0.850 |
| Intrahepatic bile duct dilatation (cm) | 1.3 (1.0, 1.6) | 1.3 (1.0, 1.6) | 1.2 (1.0, 1.6) | 0.981 |
| DWI signal (high) | 80 (76.9%) | 51 (79.7%) | 29 (72.5%) | 0.397 |
| ADC_Lesion_ (mm^2^/s) | 1.3 (1.1, 1.5) | 1.3 (1.1, 1.4) | 1.4 (1.1, 1.6) | 0.411 |
| SIR_Liver/Muscle_ | 0.9 (0.8, 2.3) | 0.9 (0.8, 1.1) | 1.0 (0.8, 1.2) | 0.434 |
| SIR_Spleen/Muscle_ | 2.0 (1.7, 2.3) | 2.0 (1.7, 2.3) | 2.1 (1.8, 2.4) | 0.326 |
| SI_Liver_ | 1.0 (0.9, 1.1) | 1.0 (0.9, 1.1) | 1.0 (0.9, 1.1) | 0.963 |
| SI_Spleen_ | 0.9 (0.8, 1.0) | 0.9 (0.8, 1.0) | 0.8 (0.7, 0.9) | 0.063 |

Note: Continuous variables with normal distribution were presented as mean ± standard deviation, using the *t* test for comparison, and those with abnormal distribution were represented as median (Q1, Q3), using Mann-Whitney *U* tests for comparisons. Categorical variables were presented as N (%) according to different levels and used chi-square tests for comparisons. All comparisons were performed between the death and the survival groups.


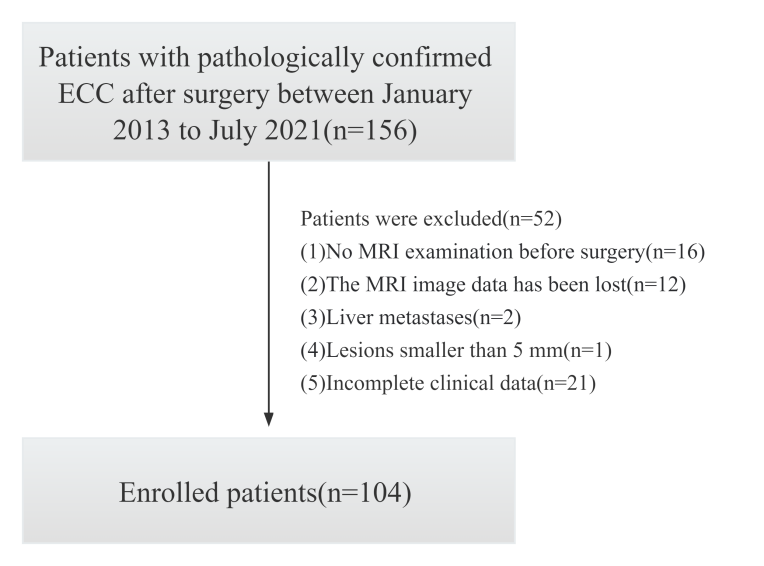


**Supplementary Figure 1. Flowchart for patient selection and exclusion criteria.**


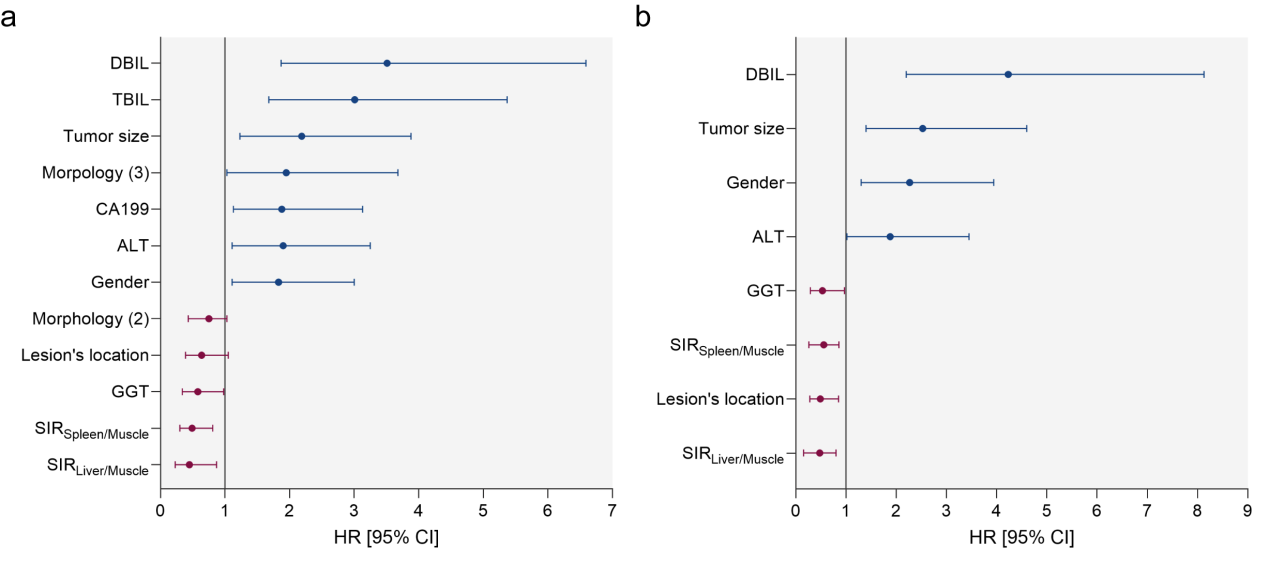


**Supplementary Figure 2. Forest plots showing the hazard ratio (HR) values of important factors in the (a) univariable analysis and (b) multivariable analysis.** The point represented the mean, and the error bar represented the 95% confidence interval (CI). The risk factors with HR values above 1 were marked in blue, while the protective factors with HR values below 1 were marked in red.


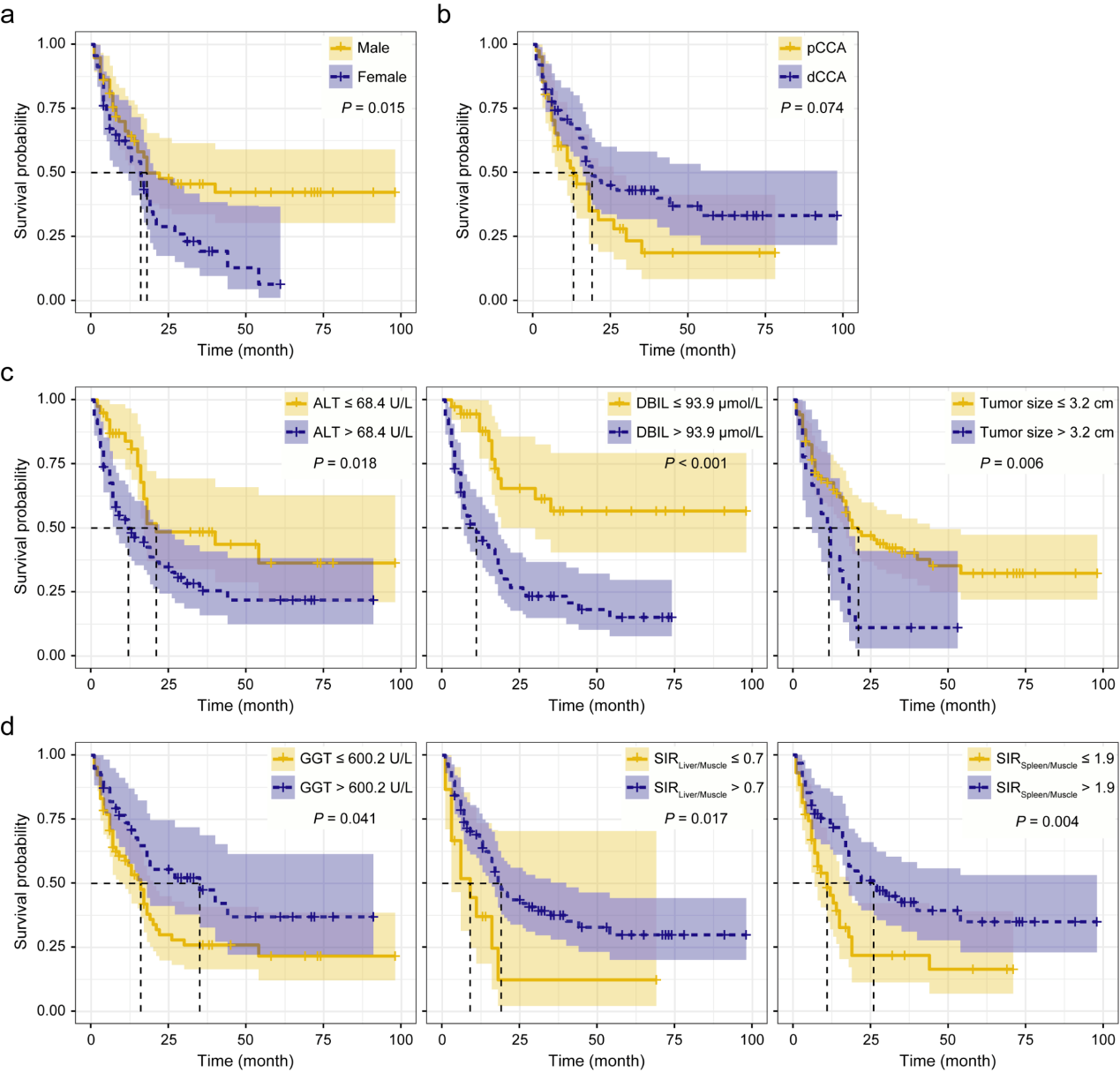


**Supplementary Figure 3. Kaplan-Meier (KM) survival curves of eight important factors in influencing the overall survival (OS). (a)** Gender and **(b)** lesion’s location were two categorical variables. **(c)** The risk continuous variables for OS, including ALT, DBIL, and tumor size. **(d)** The protective variables for OS, including GGT, SIR_Liver/Muscle_, and SIR_Spleen/Muscle_. All continuous variables were binarized by X-tile software.


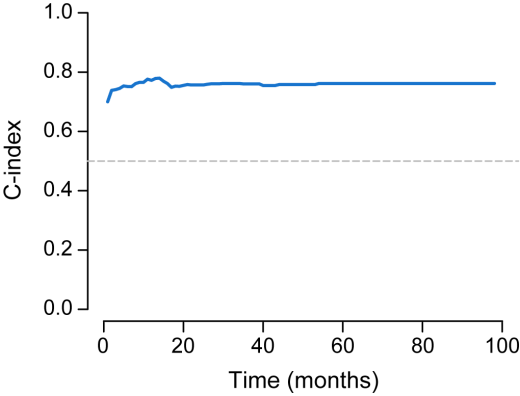


**Supplementary Figure 4. Time-dependent concordance index (C-index) curve of the COX proportional hazard model.**

**Supplementary Table 4 Quantitative metrics in cross-validation experiments.**

|  | 10-fold cross-validation of 10 times | 5-fold cross-validation of 200 times |
| --- | --- | --- |
| AUC at 1 year | 0.833 (0.719, 0.951) | 0.821 (0.752, 0.880) |
| AUC at 2 year | 0.863 (0.731, 0.952) | 0.842 (0.779, 0.899) |
| C-index at 1 year | 0.786 (0.683, 0.861) | 0.758 (0.706, 0.809) |
| C-index at 2 year | 0.755 (0.666, 0.818) | 0.738 (0.694, 0.779) |

Note: Continuous variables with abnormal distribution were represented as the median (Q1, Q3).
